# Clinical Characteristics of Necrotizing Enterocolitis in Preterm Patients With and Without Persistent Ductus Arteriosus and in Patients With Congenital Heart Disease

**DOI:** 10.3389/fped.2020.00257

**Published:** 2020-06-05

**Authors:** Sonja Diez, Lea Tielesch, Christel Weiss, Julia Halbfass, Hanna Müller, Manuel Besendörfer

**Affiliations:** ^1^Friedrich-Alexander-Universität (FAU) Erlangen-Nürnberg, Department of Surgery, Section Pediatric Surgery, University Hospital Erlangen, Erlangen, Germany; ^2^Department of Medical Statistics & Biomathematics, Medical Faculty Mannheim, Heidelberg University, Heidelberg, Germany; ^3^Friedrich-Alexander-Universität (FAU) Erlangen-Nürnberg, Pediatric Cardiology, University Hospital Erlangen, Erlangen, Germany; ^4^Friedrich-Alexander-Universität (FAU) Erlangen-Nürnberg, Neonatology and Pediatric Intensive Care, Hospital for Children and Adolescents, University Hospital Erlangen, Erlangen, Germany

**Keywords:** necrotizing enterocolitis, NEC, congenital heart disease, patent ductus arteriosus, fulminant NEC

## Abstract

**Background:** Diagnosis and management of NEC is based on clinical, radiological, and laboratory findings. Discrimination of pathogens for an improved understanding of NEC in preterm infants and NEC in infants with congenital heart disease has been previously discussed and enables evaluation of further NEC biomarkers.

**Patients and Methods:** Within a study period of 11 years (2008–2019), we identified 107 patients with a diagnosis of NEC at our primary care center. Thirty-six out of 54 patients suffering from NEC in high Bell stages who underwent surgery met inclusion criteria. These patients were classified according to their cardiac status, and analyses of clinical factors influencing NEC were conducted. Additionally, clinical factors associated with a fulminant course of NEC were examined. Univariable and multivariable analyses were performed.

**Results:** The study populations consisted of 12 preterm infants with NEC but without patent ductus arteriosus (PT-NEC), seven preterm infants with NEC and patent ductus arteriosus (PDA-NEC), and 17 infants with NEC and congenital heart disease (CHD-NEC). Blood flow in intestinal vessels was impaired in infants with PDA-NEC and CDH-NEC. Therefore, we used logistic regression to compare PDA-NEC and CDH-NEC infants with PT-NEC infants: positive bacterial culture of intraoperative swabs (*p* = 0.0199; odds ratio: 21.9) and macroscopic intestinal necrosis (*p* = 0.0033; odds ratio: 43.5) were observed more frequently in the first group. Furthermore, multiple regression analysis determined the NEC localization (*p* = 0.0243) as a significant factor correlated with a fulminant course. Compared to a NEC exclusively localized in the colon, there is a 5.8-fold increased risk of a fulminant course when the small intestine is affected and a 42-fold increase of risk when both small intestine and colon were affected.

**Conclusion:** An early diagnosis and timely surgical intervention of NEC, especially in infants with PDA and CDH may be considered to avoid major bowel necrosis (resulting in loss of intestinal tissue) and multiple operations.

## Introduction

Necrotizing enterocolitis (NEC) is a major cause of morbidity and mortality in preterm infants with a case fatality rate of 15–30% (1), leading to profound intestinal injuries and resulting in multiple long-term disabilities, such as intestinal strictures, short bowel syndrome, or neurodevelopmental delay. Incidence rates are estimated at 3–15% (2), whereas 7–20% of all NEC cases occur in term infants (3). Velazco et al. (4) identified CHD in almost 20% of NEC patients with a birth weight of more than 2,500 g. NEC prevalence in patients with congenital heart disease (CHD) is estimated at 3.7% (5). Nowadays, incidence rates are decreasing due to specialized preventive care (2). However, mortality rates remain stable.

Optimum treatment remains a challenge and requires interdisciplinary management. Decisions on appropriate empiric antibiotic regimen/strict bowel rest with gastric decompression in addition to timing of surgery are, thus, discussed individually in each case (6). In 20–40% of all cases, surgical intervention results from a lack of clinical improvement under conservative therapy. In cases of pneumoperitoneum or other evidence of intestinal perforation, surgical intervention is generally indicated and, thus, recommended as ultima ratio (7).

Pathogenesis of NEC is considered a multifactorial process. However, a central hypothesis is discussed regarding an initial insult (intestinal ischemia, bacterial overgrowth) leading to loss of intestinal mucosal integrity (6). Inflammatory processes by baseline elevation of circulating endotoxin and proinflammatory cytokines (e.g., sepsis, systemic inflammation, shock) are the possible cause of further injuries, such as bowel necrosis. In recent research, separation of pathophysiology, incidence, and mortality of NEC in CHD patients and NEC in preterm infants was brought into focus (8–10). Accordingly, impaired intestinal perfusion and reduced intestinal arterial oxygen saturation in infants with cyanotic heart disease outrank classical causes in pathogenesis and are the leading causes for its development and mortality. Patent ductus arteriosus (PDA), on the other hand, causes diastolic dysfunction, leading to heart failure, organ hypoperfusion, and oxygen deficiency in target tissues owing to the steal phenomenon, which is defined as a decreased tissue perfusion caused by diastolic backflow to areas of lower resistance despite their normal arterial oxygen saturation (3, 11). Until now, there is no explicit differentiation in literature between NEC in preterm infants with PDA and NEC in infants without PDA concerning characterization of NEC manifestation and further clinical course.

Based on this knowledge of a distinguished pathophysiology, we hypothesized that the three different patient subgroups (preterm infants without PDA, preterm infants with PDA, and infants with CDH) with advanced NEC show different clinical characteristics. The purpose of this study is to describe the manifestation and clinical course of NEC within these three different patient subgroups in order to achieve therapeutic improvement. A further goal was to develop markers indicating a fulminant course of NEC.

## Patients and Methods

### Data Collection and Inclusion Criteria

This is a retrospective study carried out on all infants with clinical signs of NEC treated in our perinatal center over a period of 11 years from January 2008 to July 2019. The study was approved by the local ethics committee in accordance with the declaration of Helsinki (1964) and its later amendments.

We collected all data for infants with the diagnosis of NEC within this period. Data were extracted from medical reports, including all clinical parameters and therapeutic measures. NEC was diagnosed and staged by modified Bell's criteria (12). The indication for surgery was based on signs of intestinal perforation in diagnostics or the infant's deterioration during conservative therapy, including antibiotic treatment and exclusive parenteral nutrition. In the latter, velocity of progression into a septic clinical picture led to indication for immediate surgery in cases of a high possibility of unstoppable intestinal necrosis and irreversible deterioration without surgery. The following criteria were included in the diagnosis: changes in blood pressure and respiratory parameters, need of fluids to maintain circulation, need for catecholamines, and advanced mechanical ventilation to achieve satisfying oxygen levels. There were no differences in surgical indication between preterm patients and patients with cardiac concomitant diseases as the same surgical team led diagnostic and therapeutic management in all patients within the whole study time.

**Figure 1:**
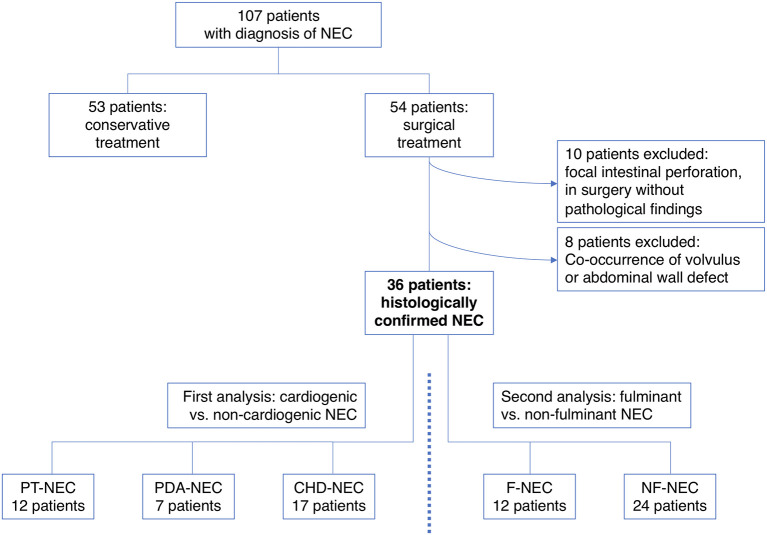
Study design and composition of study cohort.

The focus of our analysis is surgically treated patients with confirmed diagnosis due to histologic findings. Therefore, all conservatively successfully treated patients were excluded from the study. Furthermore, patients were excluded if NEC was not confirmed intraoperatively and in cases of co-occurrence with volvulus or abdominal wall defects (exact discrimination of causing pathogenesis was not possible in these cases). Figure 1 illustrates the study design and shows the allocation of patients to the different subgroups. The three different subgroups regarding cardiac status comprised preterm infants without any cardiac pathological findings, preterm infants with PDA, and infants with congenital cardiac malformations. The subgroup of cardiac malformations included all septal defects, pulmonary stenosis/atresia, transposition of great arteries, and hypoplastic left heart syndrome as well as tetralogy of Fallot, univentricular malformations, and Ebstein's disease. Congenital heart defects oftentimes consist of multiple malformations; therefore, patients were assigned to groups of their leading diagnosis. Diagnosis of reduced intestinal perfusion in patients with PDA or CHD was made on the basis of sonography and doppler. In all of these patients, an impact on intestinal diastolic flow could be seen (reduced or even reversed end-diastolic flow). In contrast, in almost all of the patients of the preterm subgroup (11/12 patients), direct or indirect signs of a sufficient intestinal perfusion could be confirmed.

The second subgroup analysis addressed the fulminant courses of NEC. Surgically treated patients were subdivided retrospectively into patients with fulminant NEC and infants with non-fulminant NEC on the basis of clinical experience as there is no consistent definition in literature. Fulminant NEC was retrospectively defined as NEC with extensive necrosis, requiring multiple surgical interventions or intestinal resection of >20 cm.

### Statistical Analysis and Structure of Analysis

Data were recorded as quantitative variables or categorical factors and analyzed according to subgroups. SAS software release 9.4 (SAS Institute Inc., Cary, NC, USA) was used for all statistical analyses.

In order to compare the three subgroups, Kruskal–Wallis tests were used for quantitative data. For qualitative data, chi-squared test or Fisher's exact test have been performed as appropriate. In the case of a statistically significant test result, comparisons between two groups were conducted using Mann–Whitney *U* test, chi-square, or Fisher's exact test, respectively, using Bonferroni correction to adjust for multiple comparisons. Furthermore, multiple logistic regression analysis was performed to compare preterm infants without PDA (PT-NEC) with patients with cardiac malformation (PDA-NEC and CHD-NEC subgroups) as well as to compare infants with fulminant NEC vs. infants with non-fulminant NEC. Backward selection was used in order to find an optimal combination of variables explaining the relevant binary outcome. In general, a test result with a *p*-value < 0.05 was considered statistically significant.

## Results

### Demographic Baseline Data

Over the study period of 11 years, we treated a total of 107 patients with NEC in our clinic. In 50% of cases, patients with NEC needed surgical intervention (*n* = 54). After exclusion of 18 patients according to exclusion criteria, all analyses were conducted for the remaining 36 patients. The study population consisted of 12 preterm infants with NEC but without patent ductus arteriosus (PT-NEC), seven preterm infants with NEC and PDA (PDA-NEC), and 17 NEC cases with CHD (CHD-NEC, see Figure 1). Baseline demographics of the included patients are shown in Table 1. Median gestational age at birth was 31.3 weeks (post-menstrual age) with a median birth weight of 1,825 g. APGAR values were measured at 1, 5, and 10 min after birth with a median of 6.0 (range: 0–10), 8.0 (range: 3–10), and 8.5 (range 3–10), respectively. Initial respiratory support using continuous positive airway pressure (CPAP) had to be applied in 19 cases (53%); primary mechanical ventilation was initiated in four newborns (11%). Nutrition was started in all infants. Fourteen patients out of 36 cases (39%) were fed with mainly human milk or human milk exclusively. The median age at NEC onset was 18.0 days of life (range: 3–105). Increased gastric residuals and abdominal distension were the initial signs of beginning NEC in a great majority of cases. Early symptoms included abdominal distension in 97% of cases (*n* = 35), whereas hematochezia (31%, *n* = 11) and abdominal erythema (17%, *n* = 6) could only be observed in a few cases.

**Table 1:** Patients' demographical data.

**Parameter**	
Sex [*n* (%)]	*n =* 36
Male	25 (69%)
Female	11 (31%)
Gestational age [weeks] [median (range)]	31.3 (23.6–41.4)
Weight at birth [g] [median (range)]	1,825 (550–3,680)
Classification in analysis subgroups [*n* (%)]	*n =* 36
PT-NEC	12 (33%)
PDA-NEC	7 (20%)
CHD-NEC	17 (47%)
Leading cardiac malformations [*n* (%)]	*n =* 17
Atrial/Ventricular septal defect	1 (3%)
Pulmonary stenosis/atresia	4 (11%)
Double inlet left ventricle	1 (3%)
Transposition of great arteries (with functionally univentricular heart)	5 (14%)
Hypoplastic left heart syndrome	3 (8%)
Tetralogy of Fallot	2 (6%)
Ebstein's disease	1 (3%)
(Postnatal) Respiratory support [*n* (%)]	*n =* 34
Spontaneous breathing	11 (31%)
Mechanical ventilation	4 (11%)
CPAP	19 (53%)
CPAP before diagnosis [*n* (%)]	*n =* 36
No	14 (39%)
Yes	22 (61%)
Postnatal infection [*n* (%)]	*n =* 35
No	20 (56%)
Yes	15 (42%)
Human milk [*n* (%)]	*n =* 36
Human milk exclusively	8 (22%)
Mainly human milk	6 (17%)
Mainly formula, but human milk included	5 (14%)
No human milk	17 (47%)
Outcome: short bowel syndrome [*n* (%)]	*n =* 36
No	29 (81%)
Yes	7 (19%)
Outcome: survival [*n* (%)]	*n =* 36
No	10 (28%)
Yes	26 (72%)

Due to the study design, high Bell stages are characteristic of the study population (12). Most patients were assigned to the group of modified Bell's stage III (*n* = 22, 61%) and IIb (*n* = 11, 31%), whereas no patient with NEC stage I/IIa was included in this analysis. In 92% of patients, pneumoperitoneum, portal venous gas, pneumatosis intestinalis, fixed dilated intestinal loops, or other generalized intestinal dilatation could be seen in either sonographic or radiological imaging diagnostics (*n* = 33). In 40% of cases, sonographic or radiological signs of an intestinal perforation could be observed. In the remaining patients (60%), immediate surgery was conducted in cases of a high possibility of unstoppable intestinal necrosis and irreversible deterioration without surgery.

The surgical approach comprised open intestinal exploration in all cases and additional intestinal resection if needed. Surgical treatment with primary peritoneal drainage was not performed in any case. In 58% of infants with fulminant NEC, diagnosis of short bowel syndrome led to long-term parenteral nutrition (seven out of 12 patients).

Cardiac malformations in our cohort are demonstrated in Table 1. Hence, 14 out of 24 patients with cardiac malformations suffered from a cyanotic congenital heart defect (58%). All patients developed NEC prior to cardiac surgery. Overall survival rate within the study population was 72% (deaths in 10 out of 36 surgically treated NEC patients).

### Analysis of Influence of Cardiac Status

#### Univariable Analysis of Clinical Factors Within Subgroups

Supplementary Table 1 demonstrates the comparison of clinical parameters between the three different NEC groups. Table 2 enhances the statistical tests by demonstrating the results of pairwise comparisons between PT-NEC infants with PDA-NEC infants, PT-NEC infants with CHD-NEC infants, and PDA-NEC infants with CHD-NEC infants in the case of significant differences between the three groups (indicated in Supplementary Table 1).

**Table 2:** Clinical characteristics of patients divided in cardiac subgroups and analyzed between two groups.

	**PT-NEC**	**CHD-NEC**		**PT-NEC**	**PDA-NEC**		**CHD-NEC**	**PDA-NEC**	
Gestational age [weeks; median (range)]	31.1 (25.3–35.6)	38.2 (26.7–41.4)	**0.0024**	31.1 (25.3–35.6)	26.9 (23.6–29.1)	**0.0060**	38.2 (26.7–41.4)	26.9 (23.6–29.1)	**0.0018**
Weight at birth [g; median (range)]	1,670 (770–2,280)	2,580 (680–3,680)	**0.0111**	1,670 (770–2,280)	840 (550–1,220)	**0.0381**	2,580 (680–3,680)	840 (550–1,220)	**0.0027**
Postnatal in-fection [n (%)]	*n =* 12	*n =* 16	**0.0162**	*n =* 12	*n =* 7	1.0000	*n =* 16	*n =* 7	**0.0321**
No	4 (33%)	14 (82%)		4 (33%)	2 (29%)		14 (82%)	2 (29%)	
Yes	8 (67%)	2 (12%)		8 (67%)	5 (71%)		2 (12%)	5 (71%)	
Prostaglandin E1 [n (%)]	*n =* 12	*n =* 17	**0.0012**	*n =* 12	*n =* 7	nn	*n =* 17	*n =* 7	**0.0177**
No	12 (100%)	6 (35%)		12 (100%)	7 (100%)		6 (35%)	7 (100%)	
Yes	0	11 (65%)		0	0		11 (65%)	0	
COX2-In-hibitors [n (%)]	*n =* 12	*n =* 17	nn	*n =* 12	*n =* 7	**0.0054**	*n =* 17	*n =* 7	**0.0015**
No	12 (100%)	17 (100%)		12 (100%)	2 (29%)		17 (100%)	2 (29%)	
Yes	0	0		0	5 (71%)		0	5 (71%)	
Fresh frozen plasma [n (%)]	*n =* 12	*n =* 17	**0.0126**	*n =* 12	*n =* 7	1.0000	*n =* 17	*n =* 7	0.0699
No	10 (83%)	5 (29%)		10 (83%)	6 (86%)		5 (29%)	6 (86%)	
Yes	2 (17%)	12 (71%)		2 (17%)	1 (14%)		12 (71%)	1 (14%)	
CPAP before diagnosis [n (%)]	*n =* 12	*n =* 17	**0.0126**	*n =* 12	*n =* 7	1.0000	*n =* 17	*n =* 7	**0.0138**
No	2 (17%)	12 (71%)		2 (17%)	0		12 (71%)	0	
Yes	10 (83%)	5 (29%)		10 (83%)	7 (100%)		5 (29%)	7 (100%)	
Proof of bacteria intra-operatively [n (%)]	*n =* 12	*n =* 17	0.0573	*n =* 12	*n =* 7	**0.0285**	*n =* 17	*n =* 7	1.0000
No	11 (92%)	8 (47%)		1 (92%)	2 (29%)		8 (47%)	2 (29%)	
Yes	1 (8%)	9 (53%)		1 (8%)	5 (71%)		9 (53%)	5 (71%)	
Age at surgery [days; median (range)]	11 (3–49)	24(3–105)	0.1872	11 (3–49)	25 (11–81)	0.0921	24 (3–105)	25 (11–81)	1.0000
Intestinal necrosis [n (%)]	*n =* 12	*n =* 17	**0.0027**	*n =* 12	*n =* 7	**0.0285**	*n =* 17	*n =* 7	1.0000
No	11 (92%)	5 (29%)		11 (92%)	2 (29%)		5 (29%)	2 (29%)	
Yes	1 (8%)	12 (71%)		1 (8%)	5 (71%)		12 (71%)	5 (71%)	
Localization of NEC [n (%)]	*n =* 12	*n =* 17	0.4740	*n =* 12	*n =* 7	0.4356	*n =* 17	*n =* 7	**0.0483**
Small bowels	7 (58%)	4 (23%)		7 (58%)	3 (43%)		4 (23%)	3 (43%)	
Colon	3 (25%)	10 (59%)		3 (25%)	0		10 (59%)	0	
Both localizations	2 (17%)	3 (18%)		2 (17%)	4 (57%)		3 (18%)	4 (57%)	
Outcome: survival [n (%)]	*n =* 12	*n =* 17	0.0573	*n =* 12	*n =* 7	1.0000	*n =* 17	*n =* 7	0.0669
No	1 (8%)	9 (53%)		1 (8%)	0		9 (53%)	0	
Yes	11 (92%)	8 (47%)		11 (92%)	7 (100%)		8 (47%)	7 (100%)	

*Tests were conducted if global analysis (Supplementary Table 1) showed statistically significant results after adjustment by Bonferroni–Holm. Statistical testing could be conducted in 12 preterm infants without patent ductus arteriosus (PT-NEC), 17 infants with congenital heart disease (CHD-NEC), and seven preterm infants with patent ductus arteriosus (PDA-NEC). Due to small numbers, statistical tests were not conductible for every constellation (nn = non nominatus). Significant results have been highlighted (bold values)*.

Infants with CDH and preterm infants with PDA showed reduced intestinal blood flow due to PDA or cardiac malformation in comparison to preterm infants without PDA as described in previous studies. Therefore, we compared CDH-NEC infants/PDA-NEC infants with PT-NEC infants using univariable analysis (Table 3).

**Table 3:** Comparison between infants with impaired intestinal perfusion (CDH-NEC, PDA-NEC) and preterm infants without PDA (PT-NEC).

	**Classification by cardiac status**		
**Parameter**	**PT-NEC**	**CHD/PDA-NEC**	***p*-value**
Sex [n (%)]	*n =* 12	*n =* 24	1.0000
Male	8 (67%)	17 (71%)	
Female	4 (33%)	7 (29%)	
Gestational age [weeks; median (range)]	31.1 (25.3–35.6)	36.3 (23.6–41.4)	0.2171
Weight at birth [g; median (range)]	1670 (770–2280)	2080 (550–3680)	0.2877
CPAP before diagnosis [n (%)]	*n =* 12	*n =* 24	0.0765
No	2 (17%)	12 (50%)	
Yes	10 (83%)	12 (50%)	
Postnatal infection [n (%)]	*n =* 12	*n =* 23	**0.0398**
No	4 (33%)	16 (67%)	
Yes	8 (67%)	7 (29%)	
Prostaglandin E1 [n (%)]	*n =* 12	*n =* 24	**0.0059**
No	12 (100%)	13 (54%)	
Yes	0	11 (46%)	
Fresh frozen plasma transfusion [n (%)]	*n =* 12	*n =* 24	**0.0314**
No	10 (83%)	11 (46%)	
Yes	2 (17%)	13 (54%)	
Catecholamines [n (%)]	*n =* 12	*n =* 24	0.2357
No	7 (58%)	9 (37%)	
Yes	5 (42%)	15 (63%)	
Human milk [n (%)]	*n =* 12	*n =* 24	0.4929
Human milk exclusively	2 (17%)	6 (25%)	
Mainly human milk	1 (8%)	5 (21%)	
Mainly formula, but human milk included	1 (8%)	4 (17%)	
No human milk	8 (67%)	9 (37%)	
CRP at diagnosis [mg/l; median (range)]	8.0 (0.6–259.0)	55.7 (0.3–239.0)	0.0674
Proof of bacteria intraoperatively [n (%)]	*n =* 12	*n =* 24	**0.0041**
No	11 (92%)	10 (42%)	
Yes	1 (8%)	14 (58%)	
Age at surgery [days; median (range)]	11 (3–49)	25 (3–105)	**0.0224**
Intestinal necrosis [n (%)]	*n =* 12	*n =* 24	**0.0004**
No	11 (92%)	7 (29%)	
Yes	1 (8%)	17 (71%)	
Intestinal perforation [n (%)]	*n =* 12	*n =* 24	0.2357
No	7 (58%)	9 (37%)	
Yes	5 (42%)	15 (63%)	
Localization of NEC [n (%)] Small bowels	*n =* 12 7 (58%)	*n =* 24 7 (29%)	
Colon	3 (25%)	10 (42%)	0.3270
Both localizations	2 (17%)	7 (29%)	
Number of needed surgeries [n (%)]	1 (1–4)	1 (1–3)	0.2808
Outcome: short bowel syndrome [n (%)]	*n =* 12	*n =* 24	0.6639
No	9 (75%)	20 (83%)	
Yes	3 (25%)	4 (17%)	
Outcome: survival [n (%)]	*n =* 12	*n =* 24	0.1149
No	1 (8%)	9 (37%)	
Yes	11 (92%)	15 (63%)	
Fulminant NEC [n (%)]	*n =* 12	*n =* 24	0.7091
No	9 (75%)	15 (63%)	
Yes	3 (25%)	9 (37%)	

*Significant results have been highlighted (bold values)*.

#### Comparison of Clinical Factors Between Infants With Impaired Intestinal Perfusion (CDH-NEC, PDA-NEC) and Preterm Infants Without PDA (PT-NEC) Using Multivariable Analysis

Logistic regression was used in order to analyze several parameters simultaneously. Detection of bacteria in intraoperatively performed swabs by bacterial culture could be seen more frequently in patients with PDA-NEC and CDH-NEC compared to preterm infants without PDA (*p* = 0.0199; odds ratio: 21.9), indicating an elevated amount of bacteria migration through the intestinal wall. Furthermore, logistic regression revealed significantly increased macroscopic intestinal necrosis (*p* = 0.0033; odds ratio: 43.5) in CDH- and PDA-NEC groups. Again, this fact additionally represents an advanced stage of NEC with macroscopic necrosis. The area under the curve (AUC) as a measure of goodness of fit is 0.913. This indicates a quite reliable statistical model.

### Analysis of Fulminant NEC

#### Univariable Analysis of Fulminant NEC

We suggest improving clinical assessment of NEC by defining fulminant NEC retrospectively in cases of multiple surgical interventions and/or an intestinal resection >20 cm in order to identify confounding variables and clinical associations with a high-risk clinical course and poor outcome. Table 4 depicts the results of univariable analysis in consideration of comparison between infants with fulminant NEC and infants with non-fulminant NEC.

**Table 4:** Comparison of infants with and without fulminant NEC.

**Parameter**	**NF-NEC**	**F-NEC**	**p-value**
Gestational age [weeks; median (range)]	34.1 (24.4–41.4)	29.5 (23.6–39.0)	0.1751
Weight at birth [g; median (range)]	1,940 (600–3,300)	1,185 (550–3,680)	0.4175
Postnatal infection [n (%)]	*n =* 23	*n =* 12	0.4109
No	12 (50%)	8 (67%)	
Yes	11 (46%)	4 (33%)	
Cardiac defect [n (%)]	*n =* 24	*n =* 12	0.4201
No cardiac defect	9 (37%)	3 (25%)	
Cardiac malformation	12 (50%)	5 (42%)	
PDA	3 (13%)	4 (33%)	
Prostaglandin E1 [n (%)]	*n =* 24	*n =* 12	0.2681
No	15 (63%)	10 (83%)	
Yes	9 (37%)	2 (17%)	
COX2-Inhibitors [n (%)]	*n =* 24	*n =* 12	**0.0336**
No	23 (96%)	8 (67%)	
Yes	1 (4%)	4 (33%)	
Catecholamines [n (%)]	*n =* 24	*n =* 12	0.8125
No	11 (46%)	5 (42%)	
Yes	13 (54%)	7 (58%)	
Human milk [n (%)]	*n =* 24	*n =* 12	0.5262
Human milk exclusively	6 (25%)	2 (17%)	
Mainly human milk	5 (21%)	1 (8%)	
Mainly formula, but human milk included	2 (8%)	3 (25%)	
No human milk	11 (46%)	6 (50%)	
X-ray pathologies [n (%)]	*n =* 18	*n =* 10	1.0000
No	1 (4%)	1 (8%)	
Yes	17 (71%)	9 (75%)	
Age at surgery [days; median (range)]	15 (3–105)	27 (7–81)	0.1350
Number of needed surgeries [n (%)]	*n =* 24	*n =* 12	0.0680
	1 (1–3)	1.5 (1–4)	
Localization NEC [n (%)]	*n =* 22	*n =* 12	**0.0144**
Small bowels	9 (37%)	5 (42%)	
Colon	12 (50%)	1 (8%)	
Both	3 (13%)	6 (50%)	
Outcome: short bowel syndrome [n (%)]	*n =* 24	*n =* 12	**<0.0001**
No	24 (100%)	5 (42%)	
Yes	0	7 (58%)	

*Significant results have been highlighted (bold values)*.

#### Multivariable Analysis of Fulminant NEC

Multiple regression analysis revealed the localization of NEC (*p* = 0.0243) and the number of surgeries (*p* = 0.0450) as most relevant factors associated with fulminant NEC. The risk of a fulminant NEC—compared to an NEC localized exclusively in the colon—is 5.8-fold increased in cases of affliction of the small intestine and 42-fold increased when both small intestine and colon are affected. This model showed an AUC of 0.847. Bowel loss and persistent intestinal failure could be seen especially in fulminant NEC cases.

## Discussion

Most recently, discrimination of NEC in preterm infants and NEC in infants with concomitant cardiac malformations has been the subject of intensive research. Previous studies have demonstrated the association of NEC with CHD, presenting controversial results on incidence, associations, and mortality (3, 8, 10, 13–16) with various population sizes and subgroup analyses. Siano et al. (8) reported that congenital heart defects could be identified as a risk factor for the incidence of NEC (OR 1.849) and increased mortality rates (OR 3.4). Lau et al. (16) associated patients with ductal dependent congenital heart disease with an elevated risk of NEC compared to their counterparts. Pathogenesis in both PT-NEC and CHD subgroups is suggested to be distinct, whereas the clinical course is equalized, and symptoms are thought to be similar (17). Focus on clinical differences in the course of diagnosis and therapy between cardiogenic NEC and classic NEC has not been scientifically discussed so far but is crucial for improvement of knowledge regarding this neonatal disease.

Although important differences in the clinical presentation of NEC in full-term infants in comparison to preterm infants have been reported (18), yet little is known about the involvement of single clinical variables on the clinical course of NEC. Therefore, the results of our retrospective study at a primary care center represent additional clinical aspects to the development of NEC. So far, only Bubberman et al. (9) have presented results on the involvement of clinical variables within a recent study, comparing 18 CHD-NEC patients and 36 selected PT-NEC patients. Results of this analysis confirm the hypothesis of a varying pathophysiology of PT-NEC and CHD-NEC patients. This is primarily explained by the fact that the colon was significantly more often involved in CHD-NEC vs. PT-NEC (86 vs. 33%, *p* = 0.03) according to mesenteric hypoxia in CHD patients. Our observations of the univariable analysis are consistent with these findings. This is mainly ascribed to previously explained pathophysiological mechanisms of blood circulation, steal phenomenon, and increased susceptibility of the colon to ischemia resulting from inferior collateral blood supply compared to other intestinal regions (19).

In contrast, we see that aggravated forms of reduced perfusions within a fulminant NEC show mainly an impact on the small intestine in the course of time. The small intestine might be influenced more extensively on the basis of its larger size in comparison to the colon. Therefore, reduction of diastolic flow might impact larger intestinal parts. Additionally, specific factors, such as decreased FOXP3+ regulatory T-cell levels or increased levels of platelet-activating factor and increased expression of its receptor in the ileum, have been identified in the development of NEC and could be specifically associated with the small intestine (20). Finally, the influence of meconium in the small intestine might be evaluated further as a basis for intestinal dilatation, impaired intestinal motility, and resulting critical perfusion. The combination of all of these specific factors and the impaired regulation of microcirculatory perfusion of the gut in preterm infants puts the small intestine with its comparably large size at a special risk in the development of NEC and its advanced courses.

Secondarily, Bubberman et al. (9) suggest further differentiation of pathophysiology of NEC between the subgroups based on a primary inflammatory process to assess disease severity. Accordingly, Bubberman et al. (10) stated an association of PT-NEC patients and elevated CRP values. However, this association remains to be controversially discussed. Within our study population, we see 13 preterm infants with postnatal infections (13/15 cases with postnatal infections), which are all early onset and show a mild clinical course. Association of elevated CRP values in the development of NEC could not be confirmed. We propose to differ strictly between postnatal infections in neonates and intraoperative positive swabs on the basis of peritonitis as presented in advanced cases of NEC in CHD patients in our study. According to the two-hit hypothesis of Garzoni et al. (21), early onset infections might be estimated as a first hit in the development of NEC in preterm infants. We, therefore, assume mild first hits in the development of NEC in our preterm subgroup with an accordingly and comparably mild clinical course of NEC. In contrast, the intestinal necrosis and proof of bacteria in the intraoperative swabs show an association with PDA- and CHD-NEC, and here, an aggravated, advanced clinical course is observable. We postulate that the two-hit hypothesis of Garzoni et al. (21) cannot be applied in these cases. It can rather be hypothesized that the continuous low intestinal perfusion, resulting in these advanced cases, may be a further model of NEC pathology, which underlines the differentiation of pathophysiology of NEC in preterm infants and in infants with PDA/CHD.

Owing to the lack of a prospective patient-control study design and the small population size of a monocentric study, our results certainly have to be interpreted cautiously. Study design was chosen on the basis of a limited, yet accurately diagnosed, study population as we only included surgically treated NECs with a histological confirmation of diagnosis. Additionally, cases were only included in which we ensured the consistency of surgeons of the department. Diagnostics and therapeutic regimen of all patients were therefore comparable. Given these known disadvantages of a small, monocentric population, we see a clear advantage in this similarity and comparability of therapy, surgical methods, and post-operative management in the described cases. This might balance the fact that no real consensus exists concerning indication, ideal timing, or type of surgical procedure, which often leads to an NEC with significant and potentially irreversible intestinal damage in cases of surgical intervention (22, 23). Whereas Bell's and Gordon's criteria are only considering clinical status of NEC patients, there are several scores regarding an indication for surgical treatment (24–27). Robinson et al. (28) propose a systematic review of surgical NEC indications and timing for surgical treatment, contributing new aspects on biomarkers and novel imaging methods (such as near-infrared spectroscopy, measuring local tissue hemoglobin oxygen saturation in patients). Multidisciplinary research is still needed to improve the diagnostic and therapeutic management and to confirm our results within larger multicenter studies.

## Conclusion

Infants with reduced intestinal blood flow due to PDA or CDH in addition to NEC showed more frequently a positive bacterial culture of intraoperative swabs and macroscopic intestinal necrosis compared to infants without these risk factors. Additionally, a fulminant course of NEC was significantly associated with NEC localization. These observations in histologically confirmed NEC cases demonstrate the hazard of reduced intestinal perfusion in the pathophysiology of NEC: An early diagnosis and a timely surgical intervention in NEC, especially in infants with PDA and CDH, may be considered to avoid short bowel syndrome and impaired outcome.

## Data Availability Statement

All datasets generated for this study are included in the article/Supplementary Files.

## Ethics Statement

The studies involving human participants were reviewed and approved by Ethics Committee, Friedrich-Alexander-Universität (FAU) Erlangen–Nürnberg. The local ethics committee did not demand informed consent due to retrospective analysis of anonymized data. Written consent for any research at the university hospital without any additional expense was provided by every participants' legal guardin/next of kin.

## Author Contributions

Initially, the concept of the study was designed and adjusted in progress (SD, MB, and HM). Patients' data were acquired and included in a database (SD and LT). Statistical analysis and interpretation of data was conducted (CW and HM), and finally, the manuscript was originally drafted by SD and revised by SD, HM, and JH. The project was supervised by MB. Submission of manuscript was conducted by corresponding author SD. All the authors contributed to the realization of the study and its publication.

## Conflict of Interest

The authors declare that the research was conducted in the absence of any commercial or financial relationships that could be construed as a potential conflict of interest.
