# Supplementary material for: Clinical Characteristics of Necrotizing Enterocolitis in Preterm Patients With and Without Persistent Ductus Arteriosus and in Patients With Congenital Heart Disease
Source: Front Pediatr. 2020 Jun 5;8:257. doi: 10.3389/fped.2020.00257 (PMC7289954; doi:10.3389/fped.2020.00257)
Supplement: Supplementary file 1 [file Data_Sheet_1.PDF]

Supplementary Table 1. Clinical characteristics of patients divided in cardiac subgroups in comparison between the three defined patient groups (univariable analysis).

	Classification by cardiac status			
**Parameter**	**PT-NEC**	**CHD-NEC**	**PDA-NEC**	**p-value**
Sex [n (%)]	n=12	n=17	n=7	1.0000
Male	8 (67%)	12 (71%)	5 (71%)	
Female	4 (33%)	5 (29%)	2 (29%)	
Mode of delivery [n (%)]	n=11	n=16	n=7	0.2168
Emergency Cesarean section	3 (25%)	1 (6%)	1 (14%)	
Cesarean section	5 (42%)	6 (35%)	5 (71%)	
Vaginal delivery	3 (25%)	9 (53%)	1 (14%)	
Umbilical cord pH value [median (range)]	7.31 (6.90 – 7.42)	7.32 (7.22 – 7.49)	7.27 (7.04 – 7.45)	0.2127
Gestational age [weeks] [median (range)]	31.1 (25.3 – 35.6)	38.2 (26.7 – 41.4)	26.9 (23.6 – 29.1)	**<0.0001**
Weight at birth [g] [median (range)]	1670 (770 – 2280)	2580 (680 – 3680)	840 (550 – 1220)	**0.0002**
Small for gestational age [n (%)]	n=12	n=15	n=7	0.3569
No	10 (83%)	9 (53%)	6 (86%)	
Yes	2 (17%)	6 (35%)	1 (14%)	
CPAP before diagnosis [n (%)]	n=12	n=17	n=7	**0.0008**
No	2 (17%)	12 (71%)	0	
Yes	10 (83%)	5 (29%)	7 (100%)	
Ventilation before diagnosis [n (%)]	n=12	n=17	n=7	0.5278
No	1 (8%)	0	0	
Yes	11 (92%)	17 (100%)	7 (100%)	
Postnatal infection [n (%)]	n=12	n=16	n=7	**0.0036**
No	4 (33%)	14 (82%)	2 (29%)	
Yes	8 (67%)	2 (12%)	5 (71%)	
Prostaglandin E1 [n (%)]	n=12	n=17	n=7	**< 0.0001**
No	12 (100%)	6 (35%)	7 (100%)	
Yes	0	11 (65%)	0	
Administration of COX2-Inhibitors [n (%)]	n=12	n=17	n=7	**< 0.0001**
No	12 (100%)	17 (100%)	2 (29%)	
Yes	0	0	5 (71%)	
Red blood cell (RBC) transfusion [n (%)]	n=12	n=17	n=7	1.0000
No	6 (50%)	8 (47%)	4 (57%)	
Yes	6 (50%)	9 (53%)	3 (43%)	
Fresh frozen plasma transfusion [n (%)]	n=12	n=17	n=7	**0.0041**
No	10 (83%)	5 (29%)	6 (86%)	
Yes	2 (17%)	12 (71%)	1 (14%)	

Platelet transfusion [n (%)]<br>No<br>Yes	n=12<br>11 (92%)<br>1 (8%)	n=17<br>10 (59%)<br>7 (41%)	n=7<br>6 (86%)<br>1 (14%)	0.1221
Catecholamines [n (%)]<br>No<br>Yes	n=12<br>7 (58%)<br>5 (42%)	n=17<br>6 (35%)<br>11 (65%)	n=7<br>3 (43%)<br>4 (57%)	0.4580
Human milk [n (%)]<br>Human milk exclusively<br>Mainly human milk<br>Mainly formula, but human milk included<br>No human milk	n=12<br>2 (17%)<br>1 (8%)<br>1 (8%)<br>8 (67%)	n=17<br>3 (18%)<br>4 (23%)<br>3 (18%)<br>7 (41%)	n=7<br>3 (43%)<br>1 (14%)<br>1 (14%)<br>2 (29%)	0.6334
Hematochezia [n (%)]<br>No<br>Yes	n=12<br>8 (67%)<br>4 (33%)	n=17<br>11 (65%)<br>6 (35%)	n=7<br>6 (86%)<br>1 (14%)	0.7185
Sonographic pathologies [n (%)]<br>No<br>Yes	n=10<br>1 (8%)<br>9 (75%)	n=15<br>1 (6%)<br>14 (82%)	n=6<br>0<br>6 (86%)	1.0000
Abdominal X-ray pathologies [n (%)]<br>No<br>Yes	n=9<br>2 (17%)<br>7 (58%)	n=14<br>0<br>14 (82%)	n=5<br>0<br>5 (71%)	0.1217
BGA at diagnosis: pH [median (range)]	7.33<br>(7.10 – 7.53)	7.33<br>(7.04 – 7.50)	7.32<br>(7.17 – 7.41)	0.9181
BGA at diagnosis: Base Excess [mmol/l] [median (range)]	-6.25<br>(-16.9 – 2.7)	-3.60<br>(-13.3 – 6.3)	-1.70<br>(-7.7 – 2.0)	0.5250
Lactate at diagnosis [mmol/l] [median (range)]	2.25<br>(1.4 – 8.8)	2.89<br>(1.0 – 14.4)	2.80<br>(1.1 – 7.7)	0.8646
Sodium at diagnosis [mmol/l] [median (range)]	131<br>(119 – 145)	132<br>(125 – 143)	127<br>(114 – 139)	0.2878
Red blood cell count at diagnosis [million mcl] [median (range)]	4.11<br>(2.43 – 5.64)	4.01<br>(2.54 – 6.28)	3.92<br>(2.80 – 4.52)	0.6701
White blood cell (WBC) count at diagnosis [n x 10^9/l] [median (range)]	5.04<br>(0.54 – 9.32)	4.68<br>(1.33 – 23.13)	5.61<br>(2.14 – 21.35)	0.7166
Platelet count at diagnosis [n/µl] [median (range)]	181<br>(22 – 314)	189<br>(51 – 571)	216<br>(80 – 413)	0.7036
CRP at diagnosis [mg/l] [median (range)]	8.0<br>(0.6 – 259.0)	49.2<br>(0 – 220.3)	98.7<br>(15.3–239.0)	0.1336
Conservative treatment >24 h [n (%)]<br>No<br>Yes	n=12<br>9 (75%)<br>3 (25%)	n=17<br>15 (88%)<br>2 (12%)	n=7<br>4 (57%)<br>3 (43%)	0.2596
Proof of bacteria intraoperatively [n (%)]<br>No<br>Yes	n=12<br>11 (92%)<br>1 (8%)	n=17<br>8 (47%)<br>9 (53%)	n=7<br>2 (29%)<br>5 (71%)	**0.0090**
Age at surgery [days]	10.5	24	25	0.0621

[median (range)]	(3 – 49)	(3 – 105)	(11 – 81)	
Intestinal necrosis [n (%)]<br>No<br>Yes	n=12<br>11 (92%)<br>1 (8%)	n=17<br>5 (29%)<br>12 (71%)	n=7<br>2 (29%)<br>5 (71%)	**0.0015**
Intestinal perforation [n (%)]<br>No<br>Yes	n=12<br>7 (58%)<br>5 (42%)	n=17<br>6 (35%)<br>11 (65%)	n=7<br>3 (43%)<br>4 (57%)	0.4580
Localization of NEC [n (%)]<br>Small bowels<br>Colon<br>Both localizations	n=12<br>7 (58%)<br>3 (25%)<br>2 (17%)	n=17<br>4 (23%)<br>10 (59%)<br>3 (18%)	n=7<br>3 (43%)<br>0<br>4 (57%)	**0.0232**
Number of needed surgeries [n (%)]	1<br>(1 – 4)	1<br>(1 – 3)	1<br>(1 – 3)	0.4970
Outcome: short bowel syndrome [n (%)]<br>No<br>Yes	n=12<br>9 (75%)<br>3 (25%)	n=17<br>16 (94%)<br>1 (6%)	n=7<br>4 (57%)<br>3 (43%)	0.1013
Outcome: survival [n (%)]<br>No<br>Yes	n=12<br>1 (8%)<br>11 (92%)	n=17<br>9 (53%)<br>8 (47%)	n=7<br>0<br>7 (100%)	**0.0057**
Fulminant NEC [n (%)]<br>No<br>Yes	n=12<br>9 (75%)<br>3 (25%)	n=17<br>12 (71%)<br>5 (29%)	n=7<br>3 (43%)<br>4 (57%)	0.4201
